# Evaluation of SNA001, a Novel Recombinant Human Thyroid Stimulating Hormone Injection, in Patients With Differentiated Thyroid Carcinoma

**DOI:** 10.3389/fendo.2020.615883

**Published:** 2021-02-17

**Authors:** Yushen Gu, Hongrong Xu, Yanling Yang, Yan Xiu, Pengcheng Hu, Min Liu, Xiangqing Wang, Jun Song, Yan Di, Jian Wang, Xiaoxia Zhang, Tao Xu, Xuening Li, Hongcheng Shi

**Affiliations:** ^1^ Department of Nuclear Medicine, Zhongshan Hospital, Fudan University, Shanghai, China; ^2^ Department of Clinical Pharmacology, Zhongshan Hospital, Fudan University, Shanghai, China; ^3^ School of Pharmacy, Yantai University, Yantai, China; ^4^ SmartNuclide Biopharma Co. Ltd, Suzhou, China

**Keywords:** rhTSH, differentiated thyroid carcinoma, whole body scan, thyroglobulin, iodine-131

## Abstract

SNA001 is a novel recombinant human thyroid stimulating hormone (rhTSH). rhTSH has long been approved in several countries to facilitate monitoring and ablation of thyroid carcinoma without hypothyroidism caused by thyroid hormone withdrawal (THW). To assess the safety, tolerance, pharmacokinetic and pharmacodynamic properties of SNA001, the two-period (SNA001 period and THW period), dose-ascending study in well-differentiated thyroid cancer (DTC) patients was designed. Three doses (0.45 mg, 0.9 mg, and 1.35 mg) of SNA001 were intramuscularly injected, twice in the SNA001 period to stimulate iodine-131 uptake and thyroglobulin (Tg) release. 24 h after the last dose of SNA001, iodine-131 (111–185 MBq) was administrated, followed by whole-body scan (WBS) 48 h later. THW period began just after SNA001 washout and lasted for about 3–6 weeks. When TSH level was above 30 mU/L, iodine-131 (111–185 MBq) was administrated, followed by a WBS and Tg detection 48 h later. Twenty-four DTC patients after thyroidectomy were enrolled; mean peak concentrations of SNA001 in 0.45, 0.9, and 1.35 mg groups were 18.5, 26.7, and 37.0 ng/ml (about 244.7, 354.2, and 489.6 mU/L) respectively, within 28–32 h after first dose of SNA001. SNA001 was metabolized in a dose-dependent manner. The results of WBS and Tg release in the SNA001 period were compared with those in the THW period. Compared to Tg level in baseline, the Tg levels in SNA001 and THW periods were increased, with 78% of subjects showing higher Tg levels in the THW period. 100% of the patients had concordant qualitative results of the scans within two periods in three groups. Symptoms of hypothyroidism were relieved in the SNA001 period compared with THW period, though there was no significant difference in most of the scale scores. There were no serious adverse events related to SNA001; the most common adverse events were gastrointestinal symptoms of mild and transient nature. Thus, SNA001 promises to be a safe and effective method to stimulate iodine-131 uptake and Tg secretion during monitoring and ablation for DTC without the disadvantages of incidental hypothyroidism.

## Introduction

Thyroid carcinoma had an estimated incidence of 567,233 new cases and 41,071 deaths in 2018, worldwide. In China, the estimated incidence in 2015 was 90,000 cases with approximately 6,800 deaths (1–3). Both estimates show that, despite the high incidence, thyroid carcinoma has high survival rates. For papillary and follicular thyroid cancer, the 10-year and 20-year survival rates surpass 90% (4). However, around 30% of patients relapse, with two-thirds of the recurrences occurring within the first decade after therapy. Tumor recurrence depends, to a great extent, on the tumor size and pathology characteristics. When total, or near-total, thyroidectomy followed by iodine-131 adjuvant and L-thyroxine therapy and regular follow-up approach are adopted, nearly 90% of the patients remain permanently free of the disease (5).

For the vast majority of differentiated thyroid carcinoma patients, especially for high-risk patients, it is recommended to undergo iodine-131 adjuvant therapy after total or near-total thyroidectomy, followed by periodic follow-up (by WBS and serum Tg measurements) (6, 7). Satisfactory radioiodine treatment and follow-up detection should be performed after TSH levels reach 30 mU/L. Recombinant human thyrotropin stimulating hormone (rhTSH) is an accepted alternative to THW to stimulate serum TSH elevation (5, 8). THW is associated with symptoms of clinical hypothyroidism in 22–25% of patients (7, 9). Conversely, rhTSH allows decreasing the diagnosis and treatment time, as well as the duration of TSH elevation, and avoiding the symptoms of hypothyroidism. Moreover, rhTSH is suitable for patients whose serum TSH does not achieve 30 mU/L after THW (10).

SNA001, a rhTSH analog, was developed by SmartNuclide Biopharma Co. Ltd (Suzhou, China). At present, Thyrogen™ (Sanofi Co.) is the unique rhTSH analog in the market, which has been approved in more than 72 countries or regions (11). However, Thyrogen™ is still not available in mainland China because of not being approved for use in China. Here, we evaluated the safety, tolerability, and pharmacokinetics of SNA001 by administering two doses to 24 DTC patients, post-surgery (if applicable), during iodine-131 adjuvant therapy. As a recombinant form of the naturally occurring protein TSH, the amino acid sequence of SNA001 is identical to the hypophysial thyrotropin. The active component of SNA001 is a heterodimeric glycoprotein expressed in Chinese hamster ovary (CHO) cells and is comprised of two non-covalently linked subunits, including alpha and beta subunits of 92 and 118 amino acids, respectively. By targeting TSH receptors in the thyroid epithelial cells, Thyrogen™ and SNA001 stimulate cyclic adenosine monophosphate (cAMP) production and upregulate the concentration of thyroid hormone (both T3 and T4) *in vivo* (preclinical data). Additionally, experiments with cynomolgus monkeys revealed linear pharmacokinetic (PK) characteristics and good safety (preclinical data). This phase I clinical trial of SNA001, conducted in 24 DTC patients, has been completed (CTR20182349) and a phase III trial (CTR20192559) is in progress. In this paper, we report the pharmacokinetics, pharmacodynamics, safety, and tolerance of SNA001, the first rhTSH analog to be available in China, in patients with differentiated thyroid carcinoma.

## Materials and Methods

### Study Conduct

This was a two-phase, single-center, open-label, dose-ascending study in patients with well-differentiated thyroid cancer (n = 24). From January 2019 to September 2019, the study was conducted at the Nuclear Medicine Department in Zhongshan Hospital, Fudan University (Shanghai, China). The study was approved by the National Medical Products Administration (NMPA, NO.2018L03170) and conducted in accordance with the protocol approved by the ethics committees of Zhongshan Hospital, Fudan University (2018-092), national authorities and international conference on harmonization—good clinical practice (ICH-GCP). All patients provided written informed consent before screening.

### Patients

Patients’ inclusion criteria were as follows: age between 18 and 70 years, presence of differentiated thyroid cancer (papillary, follicular, or mixed papillary/follicular thyroid carcinoma), total or near-total thyroidectomy performed 4 weeks prior the study, at least, thyroxin suppression or replacement therapy for more than 4 weeks, absence of distant metastasis, performance status 0–2, no major coexisting conditions.

Exclusion criteria included: currently on medications that may affect thyroid function, medical history of pituitary gland disease, having taken iodine contrast agent or medications containing iodine within 3 months before study screening, and pregnancy for women. Patients with a recent history of participation in other clinical trials were also excluded.

### Procedures

**Figure 1:**
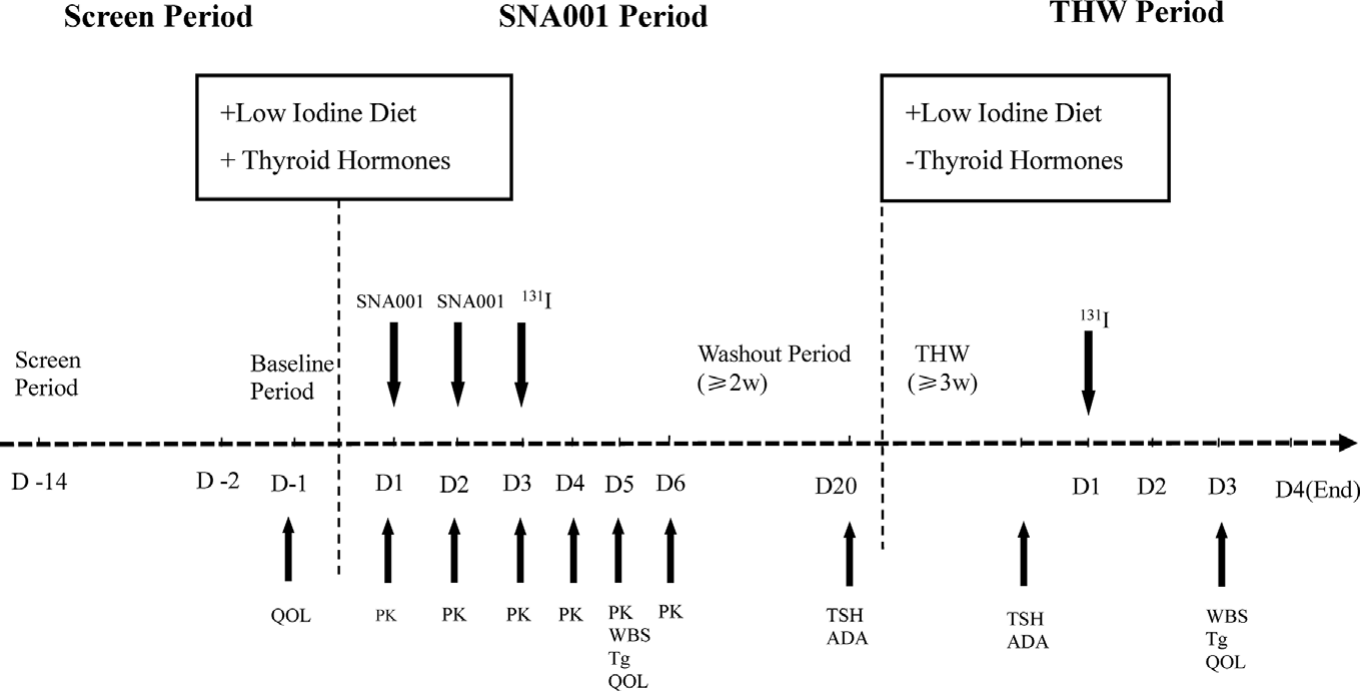
The Flow Chart of SNA001 Trial. Schematic representation of study design. Refer to the text for details.

As shown in **Figure 1**, after undergoing thyroidectomy and treatment with thyroid hormone for about 4 weeks, patients received three different dose groups of SNA001, at the first 24 h and the second 24 h. Three groups, based on SNA001 dose, were designed in this trial: 0.45, 0.9, and 1.35 mg, with eight subjects enrolled for each group. Safety and tolerability were assessed on day 6 (D6) post-administration. If the safety results of the group receiving the lowest dose met the criteria for dose ascending stopping [*i.e.*, when grade 3 or above of SNA001-related adverse events (AEs, CTCAE5.0) appeared in more than 50% of the subjects or one case of the SNA001-related serious adverse events (SAEs) occurs], the dose ascending shall be stopped. The immediately inferior dose before the one used is then considered to be the maximum tolerated dose (MTD).

After the administration of SNA001, blood samples were obtained for pharmacokinetic analyses at series of time points (showed below). Iodine-131 (111–185 MBq) was given 24 h after the final dose of SNA001. A diagnostic whole-body scan (dWBS) was performed 48 h after radioiodine administration. Then, after a washout period (≥2 weeks), thyroid hormone withdrawal (THW) resulted in the increase of endogenous TSH. In some cases, the THW period must be prolonged to 3–6 weeks to achieve TSH levels superior to 30 mU/L. During this period, the second diagnostic WBS was also performed 48 h after iodine-131 (111–185 MBq) administration.

All patients were specifically recommended to follow a low-iodine diet during the course of the study. Patients received the same diet instructions for both scans.

### Whole Body Scans

Diagnostic iodine-131 WBS was performed with a high energy collimator using a SPECT/CT system. The scanning length was set to the patient’s height (in cm) + 20 cm. The plane image was acquired as a 256 × 1,024 pixel matrix, 364 kev photopeak with a ±15% window and at a consistent scanning speed of 18 cm/min. For image construction, the antero-posterior and post-anterior projection data of patients were imported to the processing program; the color gamut as well as the size and height of the images was adjusted to ensure the images were clearly hierarchical. The obtained images were reviewed by two experienced nuclear medical physicians. Each scan was coded randomly at first, and then the two reviewers evaluated the resolution of each scan, including technical quality, normal physiological iodine uptake site, *etc*. Scans were classified according to site of uptake and number of lesions (Class 0: no uptake, Class 1: thyroid bed uptake, Class 2; uptake limited to the neck, outside thyroid bed). Lastly, the findings of the two scanning images of the same patient were evaluated to observe the consistency within the two periods (SNA001 period and THW period). Scans were considered concordant when independently assigned the same classification.

### Serum Measurement

The baseline values of serum TSH, thyroglobulin (Tg), and thyroglobulin antibody (TgAb) were measured before SNA001 administration. Tg and TgAb samples were obtained on the day of radioiodine scan during the SNA001 and THW periods.

A total of twenty-eight PK blood samples were collected for each subject. Blood samples were collected prior to administration, 30 and 60 min after administration, next hourly until 8 h post-administration, then at 12 and 24 h post-administration after the first dose of SNA001. The same scheme for sample collection was used after the second SNA001 administration, with additional samples obtained at 36, 48, 72, and 96 h post-administration of the first dose. PK analyses were performed at UP Pharma in Beijing. The validated ligand binding analysis (LBA) method, with a linear range of 0.2–25.6 ng/ml, was used to determine blood concentrations of SNA001. Non-compartmental analysis (NCA) model was used to calculate the PK parameters, using the software WinNonlin 7.0. The primary PK parameters of SNA001 were the peak concentration (C_max_), the area under the concentration–time curve (AUC) from time 0 to the time when the final concentration was measured (AUC_0–t_), and AUC was extrapolated to infinity (AUC_0–inf_). Secondary parameters included time at which C_max_ occurred (T_max_), apparent terminal elimination half-life (t_1/2_), percentage of AUC_0–inf_ extrapolation (AUC_%Extrap_), apparent terminal rate constant (*λ*_Z_), apparent volume of distribution during the terminal phase (Vz/F, where F is the fraction absorbed), mean residence time from time 0 to the time at which the lowest serum drug concentration can be detected (MRT), and the apparent clearance (CL/F) of SNA001. We used the Power model to explore the dose proportional ln(y) = *β*0 + *β*1 × log(dose), where (y) represents the PK parameter (AUC_0–t_, AUC_0–inf_, C_max_), and *β*0 represents the intercept term; *β*1 is the slope, which characterizes the degree of proportional dose–response relationship. Using the mixed effect model and setting the log (dose) as the fixed effect, the point-estimated value of slope *β*1 and its 90% confidence interval (CI) were calculated to evaluate the dose proportionality of SNA001.

Serum Tg assays were performed at the third-party central lab (KingMed Diagnostics Co., Ltd.) using an electro-chemiluminescence immunoassay (ECLIA) method employing Roche Cobas E601 instrument, with a quantitation limit of 0.1 ng/ml and a reference range for euthyroid subjects of 3.5–77ng/ml. The quantitation limit for TgAb was below 10 IU/ml, with a cut-off point of 115 IU/ml. Tg and TgAb were detected at the same time. For TgAb values above 115 IU/ml, the corresponding Tg value was regarded as inaccurate and was excluded from the Tg final compiled analysis.

Measurements for SNA001 antibodies were also performed at the third-party central lab (UP Pharma) for each patient at baseline, washout (about 3 weeks after SNA001 intramuscular injection), and the THW period before the administration of radioiodine. A validated bridging electrochemiluminescence immunoassay with a range of 50–100,000 ng/ml was used to analyze the samples.

### Quality of Life Assessment

The degree of clinical hypothyroidism was scored using the index of the 36-Item Short-Form Health Survey (SF-36) and New England Thyroid cancer specific survey profile (12–14). These survey questionnaires were completed by the patient independently, with the help of pre-training from the investigator and were evaluated at baseline, the day of the SNA001 scan and the day of the THW scan.

### Statistical Analyses

The NCA model was employed to analyze the PK parameters of SNA001 (Phoenix WinNonlin, version 7.0); C_max_ and corresponding T_max_ values were obtained directly from the concentration–time data. Quantitative data were analyzed with the software of SAS (version 9.4), and were summarized using descriptive statistics, including the number of cases, arithmetic mean, standard deviation, median, minimum, and maximum *etc*. Significant differences between groups or different periods were ascertained by two-sided test (for quantitative variables) or chi-square test (for qualitative variables).

## Results

### Patients

**Figure 2:**
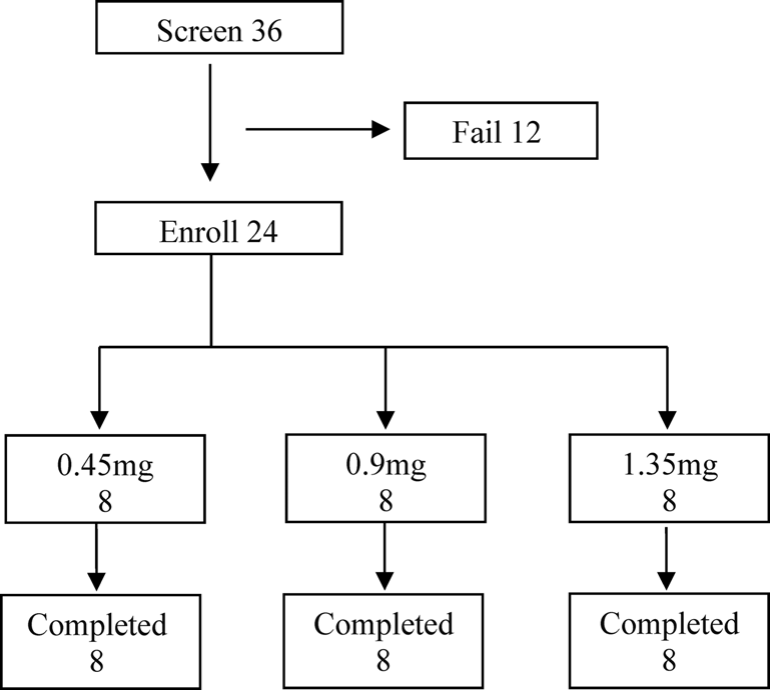
The Subjects Enrolled in the SNA001 Trial. Screening, distribution, and follow-up of study patients. Of the 12 patients who failed in screening, three patients withdrew consent; abnormal electrocardiogram was found in five patients; one patient underwent the abnormal liver function; one patient was hepatitis E positive; one patient was suspected of distant metastases, and the other one tested with high TSH. All enrolled patients completed the study.

Thirty-six subjects were screened, and twenty-four patients with well-differentiated thyroid cancer were enrolled, including 17 women and 7 men and divided in three groups of eight subjects (**Figure 2**). The median age was 48.5 year (yr), with a range of 22–69 yr. Twenty-four patients (100%) had papillary carcinoma. All of the patients underwent a total or subtotal thyroidectomy, including three patients who also underwent previous radioiodine therapy (**Table 1**). Disease staging at initial therapy is also shown in **Table 1**. There were no significant differences in the eight subjects among three groups.

**Table 1:** Characteristics of the patients enrolled in the study.

Patients	SNA001 0.45 mg (n = 8)	SNA001 0.9 mg (n = 8)	SNA001 1.35 mg (n = 8)	Overall (n = 24)
Mean age (yr)–mean (SD)	53.75(15.66)	45.13(14.89)	42.25(8.24)	47.04(13.70)
Female–N (%)	7(87.50%)	6(75.00%)	4(50.00%)	17(70.83%)
Papillary carcinoma–N (%)	8(100.00%)	8(100.00%)	8(100.00%)	24(100.00%)
Previous thyroid treatment				
Previous radioiodine therapy–N(%)	1(12.5%)	0	2(25.0%)	3(12.5%)
Total Thyroidectomy—N(%)	4(50.0%)	8(100.0%)	6(75.0%)	18(75%)
Near-total thyroidectomy–n (%)	3(37.5%)	0	0	3(12.5%)
Other previous medical history				
N (N miss)–n (%)	8(0)	8(0)	8(0)	24(0)
No – n (%)	3(37.50%)	3(37.50%)	0	6(25.00%)
Yes–n (%)	5(62.50%)	5(62.50%)	8(100.00%)	18(75.00%)
Thyroid hormone administration at baseline (μg/day)	110.42(28.96)	128.57(17.25)	165.63(116.64)	137.50(75.00)
Time from thyroidectomy to screening successfully–Mean (SD) (day)	172.38(142.96)	208.88(416.54)	201.38(259.74)	194.21(282.52)
Tumor stage–n (%)(TNM:tumor, node, metastasis)				
T1N0M0	0	0	1(12.50%)	1(4.17%)
T1N1M0	2(25.00%)	4(50.00%)	3(37.50%)	9(37.50%)
T1N1bM0	1(12.50%)	0	0	1(4.17%)
T1NxM0	0	2(25.00%)	0	2(8.33%)
T2N1M0	1(12.50%)	0	1(12.50%)	2(8.33%)
T3N1M0	2(25.00%)	2(25.00%)	3(37.50%)	7(29.17%)
T4N1M0	2(25.00%)	0	0	2(8.33%)
TSH level at baseline (mU/L) –Mean (SD)	0.47(0.63)	0.14(0.15)	0.44(0.51)	0.36(0.49)

TSH, thyrotropin.

There were no significant differences between the groups at baseline.

### Adverse Events

There were no serious adverse events related to SNA001. Among the 24 subjects enrolled in the study, the most common events were e gastrointestinal symptoms such as nausea (4.2%), vomiting (8.3%), and abdominal pain (4.2%). All adverse events and adverse reactions were mild and transient (**Table 2**).

**Table 2:** Overview of adverse events.

AE	0.45 mg (N = 8)	0.9 mg (N = 8)	1.35 mg (N = 8)	Total (N = 24)
adverse events	6(75.00%)	3(37.50%)	2(25.00%)	11(45.83%)
adverse reaction	1(12.50%)	1(12.50%)	1(12.50%)	3(12.5%)
Gastrointestinal symptoms				
nausea	0	1(12.50%)	0	1(4.17%)
vomiting	1(12.50%)	0	1(12.50%)	2(8.33%)
abdominal pain	1(12.50%)	0	0	1(4.17%)
Serious adverse event	0	0	1(12.50%)	1(4.17%)
hepatobiliary diseases				
acute cholecystitis	0	0	1(12.50%)	1(4.17%)
Serious adverse reaction	0	0	0	0
Adverse events leading to shedding	0	0	0	0

### Anti-TSH Antibody

Immunogenicity samples were obtained on baseline, washout period (about 3 weeks after SNA001 injection intramuscularly), and the THW period (before the administration of radioiodine). All immunogenicity results of the 24 patients were negative.

### Pharmacokinetics of SNA001

Baseline serum TSH concentrations were partly suppressed to 0.47 ± 0.63, 0.14 ± 0.15, 0.44 ± 0.51 mU/L in patients in the 0.45, 0.9, and 1.35 mg, respectively.

**Figure 3:**
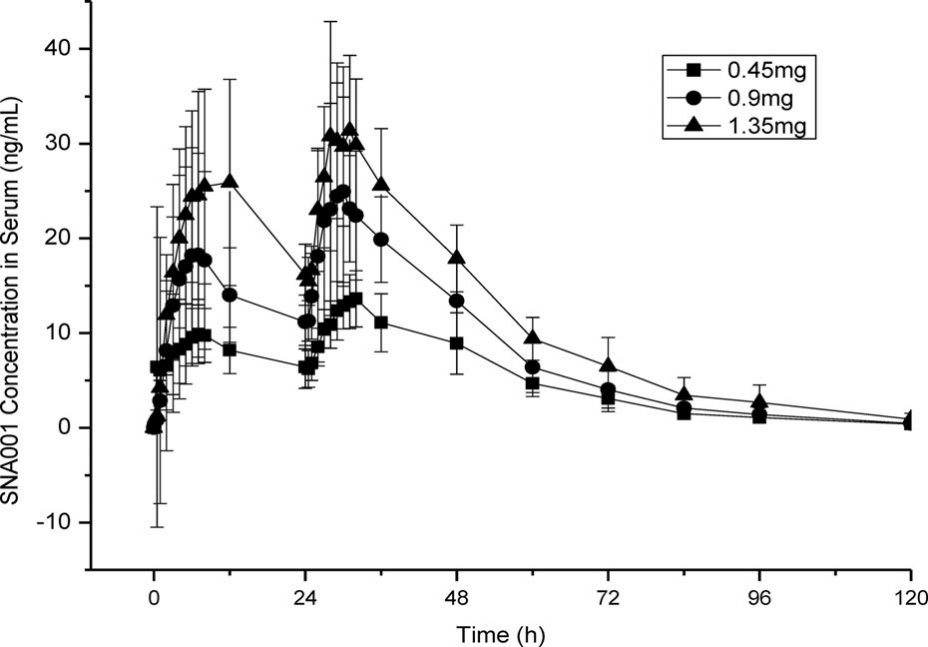
Serum SNA001 Concentration in Three Different Groups. Mean peak concentrations of SNA001 in three groups.

Both the geometric and arithmetic means of the primary PK parameters, C_max_, AUC_0–168h_ and AUC_0–inf,_ increased in a SNA001 dose-dependent manner (**Table 3**). There were no significant differences in the remaining PK parameters (Tmax, *λ*_z_, t_1/2_, V_z_/F, CL/F and MRT_0–t_) among the three groups (**Table 3**). Mean peak concentrations of rhTSH within 28–32 h after the first dose of SNA001 (about 4–8 h after the second dose) in 0.45, 0.9, and 1.35 mg groups were 18.5 ± 12.4, 26.7 ± 10.6, and 37.0 ± 10.1 ng/ml, respectively. According to the bio-specific activity of SNA001 (13.25 IU/mg, calibrated against the International Reference Preparation NIBSC 03/192), the maximal serum TSH concentrations were of 244.7, 354.2, and 489.6 mU/L, respectively for the 0.45, 0.9, and 1.35 mg groups (**Figure 3**).

**Table 3:** Pharmacokinetic parameters for SNA001.

Groups (N_cal_)	Statistic	T_max_	C_max_	AUC_0–t_	AUC_0–inf_	AUC_%Extrap_	*λ*_z_	t_1/2_	V_z_/F	CL/F	MRT_0–t_
		(h)	(ng/ml)	(ng· h/ml)	(ng· h/ml)	(%)	(1/h)	(h)	(L)	(L/h)	(h)
**0.45 mg** **(N = 8)**	Mean		18.5	630.2	641.2	1.7	0.0	16.8	35.3	1.5	37.6
	SD		12.4	148.2	152.5	0.7	0.0	2.8	9.0	0.3	4.6
	%CV		67.4	23.5	23.8	42.3	24.7	16.9	25.4	21.5	12.2
	Median	30.5	15.1	597.6	607.7	1.7	0.0	17.5	38.6	1.5	38.4
	Min	0.6	9.8	467.7	472.8	0.7	0.0	10.1	21.7	1.0	30.3
	Max	32.0	48.3	906.1	929.3	2.6	0.1	18.7	45.6	1.9	43.2
	GeoMean		16.2	616.1	626.6	1.5	0.0	16.5	34.2	1.4	37.4
**0.9 mg** **(N = 8)**	Mean		26.7	1026.6	1037.2	1.0	0.0	15.9	40.6	1.8	35.9
	SD		10.6	204.9	206.4	0.6	0.0	1.8	5.8	0.3	5.7
	%CV		39.7	20.0	19.9	60.1	10.2	11.3	14.2	18.8	15.8
	Median	31	22.5	968.8	986.9	0.9	0.0	15.3	42.2	1.8	35.8
	Min	29	19.1	808.2	814.1	0.4	0.0	14.1	30.3	1.3	25.8
	Max	36	50.5	1336.1	1341.8	2.5	0.0	19.4	48.5	2.2	45.0
	GeoMean		25.3	1009.4	1019.9	0.9	0.0	15.8	40.2	1.8	35.5
**1.35 mg** **(N = 8)**	Mean		37.0	1471.9	1496.9	1.6	0.0	17.1	45.4	1.9	36.3
	SD		10.1	263.8	267.0	1.5	0.0	3.6	9.7	0.4	5.4
	CV%		27.4	17.9	17.8	89.3	20.1	21.0	21.4	20.3	14.9
	Median	28.5	36.9	1474.8	1533.5	0.9	0.0	16.3	47.8	1.8	33.4
	Min	7	22.4	1019.7	1023.4	0.4	0.0	12.3	32.7	1.5	31.0
	Max	32	48.6	1838.5	1851.2	4.5	0.1	23.3	59.0	2.6	45.7
	GeoMean		35.7	1450.1	1474.4	1.2	0.0	16.8	44.4	1.8	36.0

SNA001 C_max_, AUC_0–168h_ and AUC_0–inf,_ in a dose-dependent manner. There were no significant differences in the remaining parameters (Tmax, λ_z_, t_1/2_, V_z_/F, CL/F and MRT_0–t_) among the three groups.

24 h after the final dose of SNA001 in the three groups, the concentrations of rhTSH decreased to 8.9 ng/ml (117.9 mU/L), 13.4 ng/ml (177.6 mU/L), and 17.9 ng/ml (237.1 mU/L) in the 0.45, 0.9, and 1.35 mg groups, respectively. 48 h after the final dose of SNA001, the concentrations of rhTSH was decreased to 3.1 ng/ml (41.1 mU/L), 4.0 ng/ml (53.0 mU/L), and 6.5 ng/ml (86.1 mU/L) in the 0.45, 0.9, and 1.35 mg groups, respectively. However, in the 0.45 mg group, only five of the eight patients (62.5%) met the criterion for TSH > 30 mU/L. In the groups of patients treated with 1.35 mg of SNA001, the TSH level rose above 30 mU/L after 72 h of administration and remained higher for 3.3 and 4 days for the low, middle, and high doses, respectively (**Table 4**).

**Table 4:** Remnant of Serum SNA001 concentration.

Timepoint	Statistic	SNA001 0.45 mg (n = 8)	SNA001 0.9 mg (n = 8)	SNA001 1.35 mg (n = 8)
24 h after the last dose	TSH level	8.9 ng/ml (about 118.0 mU/L)	13.4 ng/ml (about 177.43 mU/L)	17.9 ng/ml (about 236.8 mU/L)
	Number of the subjects (TSH > 30 mU/L)	8(100%)	8(100%)	8(100%)
48 after the last dose	TSH level	3.1 ng/ml (about 41.0 mU/L)	4.0 ng/ml (about 53.5 mU/L)	6.5 ng/ml (about 86.1 mU/L)
	Number of the subjects (TSH > 30 mU/L)	5(62.5%)	7(87.5%)	8(100%)
72 h after the last dose	TSH level	1.1 ng/ml (about 14.6 mU/L)	1.4 ng/ml (about 18.3 mU/L)	2.7 ng/ml (about 35.4 mU/L)
	Number of the subjects (TSH > 30 mU/L)	0(0.0%)	1(12.5%)	3(37.5%)
Duration days for mean TSH >30 mU/L		3 days	3 days	4 days

The concentration of TSH (ng/ml) was calculated from SNA001 PK analysis and converted to mU/L according to its bio-specific activity (13.25 IU/mg). Iodine-131 (3–5 mCi) was administrated 24 h after the final dose and followed by a WBS and Tg detection 48 h later (72 h after the last dose).

TSH >30 mU/L was observed in the subjects of 1.35 mg dose group at 72 h after the final dose, with a mean duration of 3.3 and 4 days respectively for the low, middle and high dose groups.

### Dose Linearity and Proportionality

The Power model ln (y) = *β*0 + *β*1 × log (dose) was used to evaluate the proportional dose–response relationship of SNA001. The *β*1 values for AUC_0–t_, AUC_0–inf_, C_max_ were 0.7104 [90% CI 0.4212–0.9996], 0.7719 [90% CI 0.6179–0.9259], and 0.7707 [90% CI 0.6153–0.9261] in the 0.45, 0.9, and 1.35 mg groups, respectively, indicating dose proportionality.

### Serum Tg and Tg Antibodies

**Figure 4:**
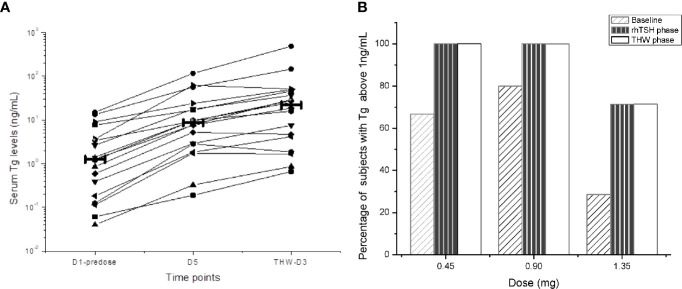
Serum Tg Level in Different Phases of Trial. Serum Tg levels at different time points. The thick solid line represents the median Tg for the group. Among them, all serum Tg levels increased at least four-fold after THW, and in 14 of the 18 subjects (78%), Tg levels were lower after SNA001 treatment than THW. However, no dose-related differences were observed in the stimulated Tg concentration. **(A)** Tg levels in different phase of trial. **(B)** Tg levels in three groups.

Tg values were considered in the analysis when the synchronized TgAb value was below the cut point, and all Tg concentration values below the quantification limit (BQL) were considered to be zero. 18 of the 24 (75%) subjects were TgAb negative. In these subjects, all Tg levels increased both in the SNA001 and THW periods compared to baseline (**Figure 4**). Among them, all serum Tg levels increased at least four-fold after THW, and in 14 of the 18 subjects (78%), Tg levels were lower after SNA001 treatment than THW. However, no dose-related differences were observed in Tg concentration.

Overall, the stimulated Tg samples of the 16 of the 18 subjects (89%) were above the cut-off value (1 ng/ml) both in D5 (SNA001 period) and in THW period. With 1 ng/ml as the cut-off value, the number of the subjects with stimulated Tg above and below the cut-off in the three dose groups was the same in the SNA001 period as in the THW period (**Figure 4**). Separately, in the two periods, the percentage of the subjects with Tg above the cut-off in the 0.45 mg dose group was 6/6 subjects (100%) and 5/5 (100%) in the 0.9 mg group. In the 1.35 mg dose group, there were 5/7 (71.4%) subjects with Tg above the cut-off and 2/7 (28.6%) subjects with Tg below the cut-off in each period.

### WBS Result

**Figure 5:**
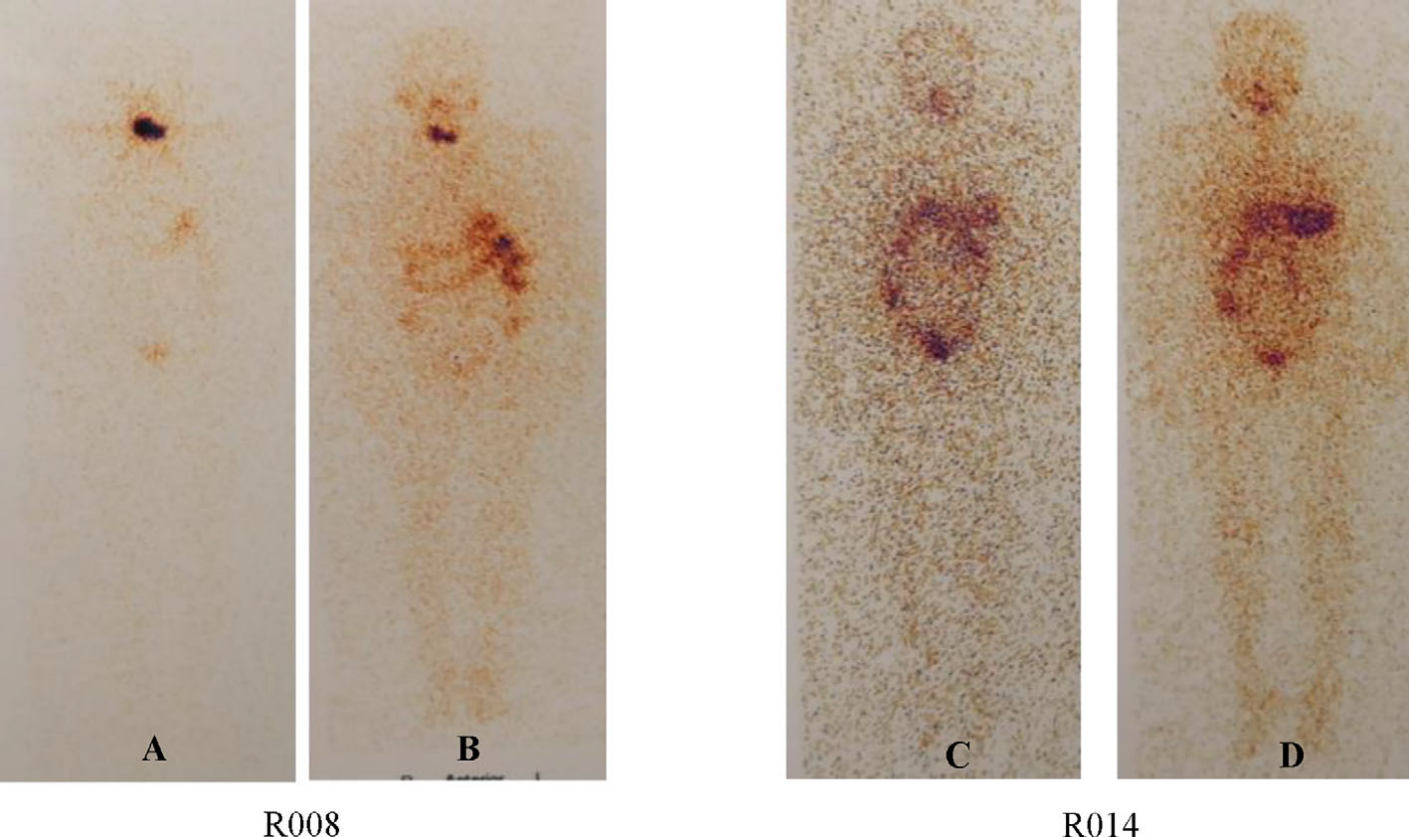
WBS in Two Different Subjects. Percentage of subjects with Tg levels above 1 ng/ml in three groups. Subjects with negative TgAb values were enrolled. **(A)** is the WBS image before radioiodine treatment. **(B)** is the WBA image after radioiodine treatment. (**C** and **D**) are the same as the former.

24/24 enrolled patients (100%) had concordant qualitative results of the scans in the two periods (**Table 5**, **Figure 5**). Among them, neck lymph node metastasis (Class 2) was found in four subjects (16.67%) in both scans, no uptake (Class 0) was found in four subjects (16.67%) in both scans, and residual thyroid bed uptake (Class 1) was seen in the remaining subjects (66.7%).

**Table 5:** WBS between SNA001 and THW periods.

WBS	Number (%)					
	0.45 mg (N = 8)		0.9 mg (N = 8)		1.35 mg (N = 8)	
	SNA001	THW	SNA001	THW	SNA001	THW
Class 0	1(12.5%)	1(12.5%)	1(12.5%)	1(12.5%)	2(25%)	2(25%)
Class 1	5(62.5%)	5(62.5%)	6(75%)	6(75%)	5(62.5%)	5(62.5%)
Class 2	2(25%)	2(25%)	1(12.5%)	1(12.5%)	1(12.5%)	1(12.5%)
Concordant	8(100%)		8(100%)		8(100%)	
Discordant	0 (0%)		0(0%)		0(0%)	

Diagnostic iodine-131 WBS was performed with SPECT/CT system.

THW: thyroid hormone withdrawn.

Class 0 means no uptake, Class 1 means Thyroid bed uptake; Class 2 means uptake limited to neck (outside thyroid bed).

### Combined Analysis of rhTSH-StimulatedTg and WBS

Eighteen subjects with negative TgAb values were enrolled for the combined analysis. Taking the scan result of THW period of each subject as the real state of the disease, the disease detection rates in the patients with thyroid bed uptake or other uptake limited to neck (Class 1 and Class 2) after THW were 83.4% (five of six), 100% (five of five), and 57.1% (four of seven) of the patients after 0.45, 0.9, and 1.35 mg of SNA001 administration, respectively using the ≥1 ng/ml Tg cut-off values **(** **Table 6** **)**. As for no uptake (Class 0) scans, the proportion was 0% (zero of six), 0% (zero of five), and 14.3% (one of seven) for SNA001 0.45, 0.9, and 1.35 mg groups respectively with Tg <1 ng/ml as the cut-off. Hence, for the combined analysis of Tg levels and scans, the overall concordant ratios were 83.4% (five of six), 100% (five of five), and 71.4% (five of seven) for the 0.45, 0.9, and 1.35 mg groups of SNA001 administration, respectively.

**Table 6:** Combined analysis of Tg levels and scans.

Tg (ng/ml)	WBS Grade	Number* (%)		
		SNA001 0.45 mg (N = 6)	SNA001 0.9 mg (N = 5)	SNA001 1.35 mg (N = 7)
≥1	Class 1	4(66.7%)	4(80%)	4(57.1%)
	Class 2	1(16.7%)	1(20%)	0(0%)
	Class 0	1(16.7%)	0(0%)	1(14.3%)
<1	Class 1	0(0%)	0(0%)	1(14.3%)
	Class 2	0(0%)	0(0%)	0(0%)
	Class 0	0(0%)	0(0%)	1(14.3%)
Concordant (rhTSH stimulated Tg *vs* THW scans)		5(83.4%)	5(100%)	5(71.4%)

*This category includes only patients who were negative for thyroglobulin antibody.

• A total of 18 patients were enrolled for the combined analysis. The WBS result of THW period of each subject was taken as the real state of the disease. Scans were classified according   to site of uptake and number of lesions (Class 0, Class 1, and Class 2, as previously described). Tg was evaluated using the 1 ng/ml as cut-off value.

• Tg levels were considered to be concordant in one the following options:

➢For Tg levels ≥1 ng/ml and corresponding to THW–WBS findings of class 1 or class 2;

➢Tg <1 ng/ml and the corresponding THW–WBS findings of class 0. The overall concordant ratios (rhTSH stimulated Tg vs THW scans) were 83.4, 100, 71.4 for the 0.45, 0.9, and 1.35 mg groups of SNA001 respectively.

### Quality of Life

There were no obvious differences on the general function between two periods except for symptoms of hypothyroidism. Seventeen symptoms were listed in a specific questionnaire for thyroid cancer. Except for “weight gain” and “constipation”, there was no significant difference (p > 0.05) for most comparison; even so, except for “changes in menstrual cycle” and “fatigue”, the thyroid cancer-specific survey showed a lower percentage of the patients with symptoms of hypothyroidism in the SNA001 period compared with the THW period **(** **Table 7** **)**. In fact, half of the patients (the proportion about 50%) have three or more symptoms in the SNA001 and THW periods.

**Table 7:** Symptoms during the rhTSH and THW periods.

Symptom*	rhTSH (n = 24)	THW (n = 24)	risk difference^#^	95%CI	P value^&^
	percent		percentage points		
Anxiety	8.3%	25.0%	−16.7%	−16.67 ± 20.55	0.121
Changes in menstrual cycle	8.3%	4.2%	4.2%	4.17 ± 13.65	0.605
Constipation	4.2%	25.0%	−20.8%	−20.83 ± 19.08	0.041
Depression	4.2%	8.3%	−4.2%	−4.17 ± 13.65	0.551
Increased sensitivity to cold temperatures	20.8%	25.0%	−4.2%	−4.17 ± 23.75	0.731
Difficulty concentrating	16.7%	20.8%	−4.2%	−4.17 ± 22.05	0.712
Dry skin	20.8%	25.0%	−4.2%	−4.17 ± 23.75	0.731
Fatigue	41.7%	33.3%	8.3%	8.33 ± 27.29	0.551
Hoarseness	25.0%	29.2%	−4.2%	−4.17 ± 25.12	0.745
Mood swings	20.8%	33.3%	−12.5%	−12.50 ± 24.89	0.330
Puffy face and hands	4.2%	16.7%	−12.5%	−12.50 ± 16.92	0.156
Reduced sexual activity	20.8%	25.0%	−4.2%	−4.17 ± 23.75	0.731
Sleep disturbance	20.8%	25.0%	−4.2%	−4.17 ± 23.75	0.731
Weight gain	12.5%	54.2%	−41.7%	−41.67 ± 23.93	0.002
Difficulty in performing usual activities at home	8.3%	25.0%	−16.7%	−16.67 ± 20.55	0.121
Difficulty in taking care of children at home ^△^	12.5%	12.5%	0.0%	0.00 ± 18.71	0.876
Difficulty in performing usual activities at work ^△^	4.2%	8.3%	−4.2%	−4.17 ± 13.65	0.605

*All symptoms were reported in a thyroid cancer specific questionnaire, which was completed on the day of WBS in SNA001 and THW periods, respectively. The choices on the questionnaire were divided into five grades according to the severity of symptoms. Data shown are for patients who described their symptoms as either “sometimes”, “moderately”, or “a lot”.

^#^A negative difference indicates that patients receiving SNA001 had better scores for symptoms or quality of life than did those undergoing THW. 95% confidence intervals were used to allow for multiple comparisons.

^&^P values were calculated by chi-square test.

^△Δ^The questions of “Difficulty in taking care of children at home” and “Difficulty in performing usual activities at work” were not available for all subjects; thus the number of patients being evaluated for these two questions was not 24 (nine and ten for the former question in rhTSH and THW periods; seven and eight for the latter question in rhTSH and THW periods).

## Discussion

The first rhTSH analog (Thyrogen™) was approved by the FDA in 1998 with the indications for use in postoperative adjunctive diagnosis in DTC patients and then for radioiodine ablation of thyroid tissue remnants in DTC patients. Currently, Thyrogen™ is on the market in 72 countries or regions (11). SNA001 is the first rhTSH analog developed in China, for which a clinical trial was conducted in a Chinese population.

Compared with the clinical trials of Thyrogen™, this trial of SNA001 provided more detailed PK data and explored a lower dose (0.45 mg) in patients with DTC. Our results might form the foundation for possible indications in the future, such as radioactive iodine (RAI) therapy for distant metastatic thyroid cancer. Moreover, more patients than the previous study at medium risk participated due to the population changed since the ATA 2015 guidelines were issued (7), which is more conforming to the current clinical status.

In this study, only three of the 24 subjects showed adverse reactions, and the types of adverse reactions were mainly mild gastrointestinal symptoms such as nausea (4.2%), vomiting (8.3%), abdominal pain (4.2%), similar with Thyrogen™ (10). There were no serious adverse reactions associated with SNA001, and all enrolled subjects completed the entire clinical trial. In addition, immunogenicity tests were negative for all enrolled subjects.

After SNA001 injection, TSH was significantly increased in all subjects and above 30 mU/L before the administration of iodine-131, meeting the requirements of the ATA guidelines for adjuvant diagnosis or RAI (7). C_max_ and AUC_0–∞_ of SNA001 in the 0.9 mg dose group were 26.7 ng/ml and 1,037.2 ng×h/ml, while the C_max_ and AUC_0–∞_ of Thyrogen™ at the same dose were 240.8 mU/L and 11,414.5 mU/L respectively (15). After converting according to the specific activity of Thyrogen™ (0.9 mg/10 IU) (16), the C_max_ and AUC_0–∞_ for Thyrogen™ were about 21.7 ng/ml and 1,027.4 ng×h/ml. As shown in the text, we regard it is necessary to monitor the remnant level of TSH at different time points and to analysis the duration days for mean TSH above 30 mU/L. After 12 h of administration of iodine-131, the TSH levels of the subjects in the groups of 0.9 and 1.35 mg SNA001 were maintained above 30 mU/L. Although no evidence supports how long high levels of TSH should be maintained for the treatment with RAI, lower doses are not recommended for clinical use to avoid individual differences which may lead to low TSH. This study indicates 0.9 mg as the optimal SNA001 dose, which might form the foundation for a follow-up phase III confirmatory clinical trial.

Among the 18 subjects with negative TgAb results, taking 1 ng/ml as the cut-off of Tg, SNA001 period and THW period has a consistent diagnosis rate of 100%, regardless of above or below the cut-off. The Tg level within the SNA001 period was lower than that of the THW, which was consistent with previous studies (17). The qualitative results of WBS in the two periods were all consistent and similar to previous studies (18), which had showed that there was no significant difference between rhTSH and THW methods in postoperative diagnosis and monitoring of DTC patients, both approaches being effective. In the previous studies (16), the combination of Tg and WBS after rhTSH administration identified 100% metastasis and successfully identified 93% of patients with residual thyroid tissue, while only 52% of patients with residual thyroid tissue could be identified by Tg alone. In this study, Tg alone successfully identified 92% of patients with residual thyroid tissue. We hypothesize that the standards of Tg detections might be different between studies. For the previous detection, the limit of detection sensitivity was 0.2 ng/ml with a reference range of 3–40 ng/ml, while in the current study, the limit is 0.1 ng/ml with a normal range of 3.5–77 ng/ml. Furthermore, in previous studies, the cut-off of Tg was 2 ng/ml, superior to the cut-off of 1 ng/ml recommended by current guidelines. Finally, we propose that the surgical methods, WBS instrument precision and measurement methods may also affect the final results during the duration of the study.

We hypothesized that higher TSH in subjects may contribute to more efficient iodine uptake in residual thyroid tissue or metastases after administration of SNA001 (15, 17). Some literature suggests that the success rate of RAI was higher for TSH levels above 90 mU/L than below (18). However, the effect of TSH levels on RAI efficiency is controversial, with data suggesting a poor correlation between the two factors (19, 20). Still, there was a reduced iodine uptake in the abdomen and kidneys during the SNA001 period compared to the THW period, which may lead to increased concentration of iodine in the sites of residual thyroid tissues and metastases (21, 22). In addition, other evidence suggests that after rhTSH injection, iodine clearance is faster than THW (23), which may lead to a decrease in iodine retention in the background. Finally, the effect caused by low dose iodine-131 in the SNA001 administration period may lead to the decrease of iodine uptake capacity during the THW period. Some studies reported that the effect appeared even after the administration of low dose iodine-131 (24–26). However, the findings are still arguable, with studies showing that the effect often happened after the high diagnostic iodine dose (27, 28).

In this study we included an evaluation of the quality of life of the patients using the SF-36 and the New England thyroid cancer-specific questionnaire. Although there were no significant differences in most of the scale scores, the differences in quality of life (QOL) were visible. Some symptoms (such as constipation, anxiety, puffy face and hands, weight gain and so on) were mild in the SNA001 than in THW period, which was similar to the result in previous studies of Thyrogen™ (14). Moreover, most of the subjects (87.5%) enrolled in our study underwent thyroidectomy within 2 years; they continued to take thyroid hormone after surgery and went through rhTSH period before the THW period, which may lead to the difference in quality of life scores which was not obvious. All these details might lead to the inconspicuous difference in QOL score. Finally, subject age and education degree may affect the comprehension of QOL questionnaire. Although the two-phase study did not provide a good result of QOL as the randomized controlled study, the differences in symptoms can also provide a reference for subsequent phase III clinical trials. Furthermore, we propose that subject training should be strengthened with inclusion of standards to improve the quality of the survey in phase III clinical trials.

Additional limitations of this study include the small size of the sample and the lack of quantitative evaluation of WBS images (such as number of locations with uptake foci). Furthermore, all the studies were done in a single center with a lack of sample diversity.

From this study, we concluded that the use of SNA001, produced in China, is effective and safe in the postoperative diagnosis of patients with differentiated thyroid cancer, and its effect is consistent with THW. Furthermore, there was an increased uptake of iodine by thyroid tissues in SNA001 *versus* THW. On the other hand, the hypothyroidism symptoms caused by thyroid hormone withdrawal disappeared with SNA001 treatment.

## Data Availability Statement

The raw data supporting the conclusions of this article will be made available by the authors, without undue reservation.

## Ethics Statement

The studies involving human participants were reviewed and approved by the Zhongshan Hospital, Fudan University (2018-092). The patients/participants provided their written informed consent to participate in this study.

## Author Contributions

TX is the corresponding author. XZ is responsible for the operation of the clinical trial. YY and YD have equal contribution to writing. All authors listed have made a substantial, direct, and intellectual contribution to the work and approved it for publication.

## Conflict of Interest

TX, XZ, YY, and YD were employed by company SmartNuclide Biopharma Co. Ltd.

The remaining authors declare that the research was conducted in the absence of any commercial or financial relationships that could be construed as a potential conflict of interest.

The authors declare that this study received funding from SmartNuclide Biopharma Co. Ltd. The funder had the following involvement with the study: design, decision to publish, and preparation of the manuscript.

## References

[1] Bray F Ferlay J Soerjomataram I Siegel RL Torre LA Jemal A. Global cancer statistics 2018: GLOBOCAN estimates of incidence and mortality worldwide for 36 cancers in 185 countries. CA: A Cancer J Clin (2018) 68:394–424. 10.3322/caac.21492 30207593

[2] Chen W Zheng R Baade PD Zhang S Zeng H Bray F. Cancer statistics in China, 2015. CA: Cancer J Clin (2016) 66:115–32. 10.3322/caac.21338 26808342

[3] Vecchia C Malvezzi M Bosetti C Garavello W Bertuccio P Levi F. Thyroid cancer mortality and incidence: a global overview. Int J Cancer (2015) 136:2187–95. 10.1002/ijc.29251 25284703

[4] Mazzaferri EL Kloos RT. Current approaches to primary therapy for papillary and follicular thyroid cancer. J Clin Endocrinol Metab (2001) 86:1447–63. 10.1210/jcem.86.4.7407 11297567

[5] Mazzaferri EL. An overview of the management of papillary and follicular thyroid carcinoma. Thyroid (1999) 9:421–7. 10.1089/thy.1999.9.421 10365671

[6] Guidelines for patients with Thyroid Nodules and Differentiated Thyroid Cancer thyroid nodule and differentiated thyroid cancer in China. (2012).

[7] Haugen BR Alexander EK Bible KC Doherty GM Mandel SJ Nikiforov YE. 2015 American Thyroid Association Management Guidelines for Adult Patients with Thyroid Nodules and Differentiated Thyroid Cancer: The American Thyroid Association Guidelines Task Force on Thyroid Nodules and Differentiated Thyroid Cancer. Thyroid Off J Am Thyroid Assoc (2016) 26:1–133. 10.1089/thy.2015.0020 PMC4739132 26462967

[8] Guidelines for ^131^I treatment of differentiated thyroid cancer (Version 2014). Chin J Nucl Med Mol Imaging (2014) 34:264–78.

[9] Pizzorno JE Murray MT Joiner-Bey ND. The Clinician’s Handbook of Natural Medicine, 3rd ed. (2016).

[10] Thyrogen: Full Prescribing Information @ FDA. (2017). https://www.accessdata.fda.gov/drugsatfda_docs/label/2017/020898s060lbl.pdf.

[11] DRUG REPORT of Thyrotropin Alpha @ Thomson Reuters Cortellis (2017).

[12] Li L Wang HM Shen Y. Chinese SF-36 Health Survey: translation, cultural adaptation, validation, and normalisation. J Epidemiol Community Health (2003) 57:259–63. 10.1136/jech.57.4.259 PMC1732425 12646540

[13] Ware JE Jr Kosinski M Bayliss MS McHorney CA Rogers WH Raczek A. Comparison of methods for the scoring and statistical analysis of SF-36 health profile and summary measures: summary of results from the Medical Outcomes Study. MedCare 33:AS264–79. 10.1097/00005650-199504000-00010 7723455

[14] Mallick U Harmer C Yap B Wadsley J Clarke S Moss L. Ablation with low-dose radioiodine and thyrotropin alfa in thyroid cancer. N Engl J Med (2012) 366:1674–85. 10.1056/NEJMoa1109589 22551128

[15] Thyrogen: Pharmacologyreview @ FDA.

[16] Thyrogen: Full Prescribing Information @ PMDA. (2008). https://www.pmda.go.jp/drugs/2008/. P200800048/index.html.

[17] Haugen BR Pacini F Reiners C Schlumberger M Ladenson PW Sherman SI. A Comparison of Recombinant Human Thyrotropin and Thyroid Hormone Withdrawal for the Detection of Thyroid Remnant or Cancer. J Clin Endocrinol Metab (1999) 84:3877–85. 10.1210/jc.84.11.3877 10566623

[18] Zhao T Liang J Guo ZQ Li TJ Lin YS. In patients with low- to intermediate-risk thyroid cancer, a preablative thyrotropin level of 30 μIU/mL is not adequate to achieve better response to ^131^I therapy. Clin Nucl Med (2016) 41:454–8. 10.1097/RLU.0000000000001167 26914559

[19] Sánchez R Espinosa-de-los-Monteros AL Mendoza V Brea E Hernández I Sosa E. Adequate Thyroid-Stimulating Hormone Levels After Levothyroxine Discontinuation in the Follow-Up of Patients with Well-Differentiated Thyroid Carcinoma. Arch Med Res (2002) 33:478–81. 10.1016/S0188-4409(02)00394-6 12459319

[20] Serhal D II Nasrallah MP Arafah BM. Rapid Rise in Serum Thyrotropin Concentrations after Thyroidectomy or Withdrawal of Suppressive Thyroxine Therapy in Preparation for Radioactive Iodine Administration to Patients with Differentiated Thyroid Cancer. J Clin Endocrinol Metab (2004) 89:3285–9. 10.1210/jc.2003-031139 15240604

[21] Cho YY Kim SK Jung JH Hahm JR Kim TH Chung JH. Long-term outcomes of renal function after radioactive iodine therapy for thyroid cancer according to preparation method: thyroid hormone withdrawal vs. recombinant human thyrotropin. Endocrinen (2019) 64:293–8. 10.1007/s12020-018-1807-x 30471053

[22] Campennì A Amato E Laudicella R Alibrandi A Cardile D Pignata SA. Recombinant human thyrotropin (rhTSH) versus Levo-thyroxine withdrawal in radioiodine therapy of differentiated thyroid cancer patients: differences in abdominal absorbed dose. Endocrine (2019) 65:132–7. 10.1007/s12020-019-01897-x 30875058

[23] Kairemo K Kangasmäki A Bom HS. Comparison of I-131 Biokinetics after Recombinant Human TSH Stimulation and Thyroid Hormone Withdrawal Measured by External Detector in Patients with Differentiated Thyroid Cancer. Chonnam Med J (2019) 55:20–4. 10.4068/cmj.2019.55.1.20 PMC6351326 30740336

[24] Schroeder PR Haugen BR Pacini F Reiners C Schlumberger M Sherman SI. A comparison of short-term changes in health-related quality of life in thyroid carcinoma patients undergoing diagnostic evaluation with recombinant human thyrotropin compared with thyroid hormone withdrawal. J Clin Endocrinol Metab (2006) 91:878–84. 10.1210/jc.2005-2064 16394083

[25] Park HM Perkins OW Edmondson JW Schnute RB Manatunga A. Influence of diagnostic radioiodines on the uptake of ablative dose of iodine-131. Thyroid (1994) 4:49–54. 10.1089/thy.1994.4.49 8054861

[26] Lassmann M Luster M Hänscheid H Reiners C. Impact of ^131^I diagnostic activities on the biokinetics of thyroid remnants. J Nucl Med (2004) 45:619–25. 15073258

[27] Morris LF Waxman AD Braunstein GD. The nonimpact of thyroid stunning: remnant ablation rates in ^131^I-scanned and nonscanned individuals. J Clin Endocrinol Metab (2001) 86:3507–11. 10.1210/jcem.86.8.7717 11502771

[28] Park HM Park YH Zhou XH. Detection of thyroid remnant/ metastasis without stunning: an ongoing dilemma. Thyroid (1997) 7:277–80. 10.1089/thy.1997.7.277 9133700

